# Unlocking the out-of-plane dimension for photonic bound states in the continuum to achieve maximum optical chirality

**DOI:** 10.1038/s41377-023-01295-z

**Published:** 2023-10-12

**Authors:** Lucca Kühner, Fedja J. Wendisch, Alexander A. Antonov, Johannes Bürger, Ludwig Hüttenhofer, Leonardo de S. Menezes, Stefan A. Maier, Maxim V. Gorkunov, Yuri Kivshar, Andreas Tittl

**Affiliations:** 1 Chair in Hybrid Nanosystems, Nanoinstitute Munich, and Center for NanoScience, Faculty of Physics, Ludwig-Maximilians-University Munich, Königinstrasse 10, 80539 München, Germany; 2 Shubnikov Institute of Crystallography, FSRC “Crystallography and Photonics”, Russian Academy of Sciences, Moscow, 119333 Russia; 3 Departamento de Física, Universidade Federal de Pernambuco, 50670-901 Recife, Pernambuco Brazil; 4 School of Physics and Astronomy, Monash University, Wellington Rd, Clayton, VIC 3800 Australia; 5 The Blackett Laboratory, Department of Physics, Imperial College London, London, SW7 2AZ UK; 6 Nonlinear Physics Centre, Research School of Physics, Australian National University, Canberra, ACT 2601 Australia

**Keywords:** Metamaterials, Nanophotonics and plasmonics

## Abstract

The realization of lossless metasurfaces with true chirality crucially requires the fabrication of three-dimensional structures, constraining experimental feasibility and hampering practical implementations. Even though the three-dimensional assembly of metallic nanostructures has been demonstrated previously, the resulting plasmonic resonances suffer from high intrinsic and radiative losses. The concept of photonic bound states in the continuum (BICs) is instrumental for tailoring radiative losses in diverse geometries, especially when implemented using lossless dielectrics, but applications have so far been limited to planar structures. Here, we introduce a novel nanofabrication approach to unlock the height of individual resonators within all-dielectric metasurfaces as an accessible parameter for the efficient control of resonance features and nanophotonic functionalities. In particular, we realize out-of-plane symmetry breaking in quasi-BIC metasurfaces and leverage this design degree of freedom to demonstrate an optical all-dielectric quasi-BIC metasurface with maximum intrinsic chirality that responds selectively to light of a particular circular polarization depending on the structural handedness. Our experimental results not only open a new paradigm for all-dielectric BICs and chiral nanophotonics, but also promise advances in the realization of efficient generation of optical angular momentum, holographic metasurfaces, and parity-time symmetry-broken optical systems.

## Abstract (web summary)

We unlock the height as accessible parameter of all-dielectric metasurfaces, allowing polarization-selective interaction of their high-Q eigenstates with light of different helicity and promoting ultra-narrow maximally chiral optical resonances.

## Graphical abstract

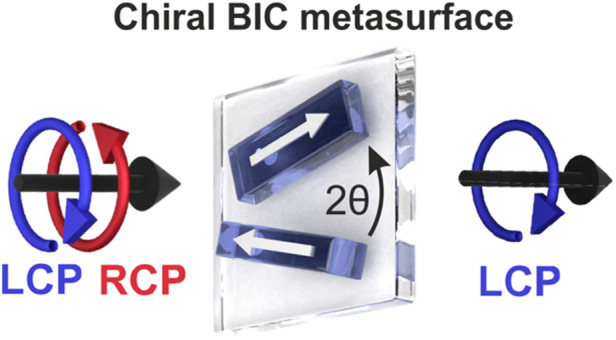

## Introduction

Controlling the interaction of different polarization states of light with matter has been a fundamental aim of optics, covering the gamut from fundamental science^1^ to practical technological applications^2^. Metasurfaces composed of resonant subwavelength building blocks with tailored optical properties have significantly advanced the capabilities for controlling light on the nanoscale^3^, launching breakthrough applications in diverse fields including localized high-harmonic generation^4,5^, ultrathin optical elements^6,7^, and biomolecular sensing^8–10^. In recent years, two major developments have underpinned the rapid progress in metasurface technology and its applications: (i) a transition from traditional plasmonic resonator geometries to all-dielectric materials to overcome Ohmic losses, and (ii) the utilization of the emerging physical concept of photonic quasi-bound states in the continuum (qBICs), which provides versatile control over the radiative losses in nanophotonic systems^11–14^. Combining these advances, all-dielectric qBIC-driven metasurfaces have delivered ultrasharp resonances with high values of the quality factors (*Q* factors)^4,15^, broad spectral tunability^9,16^, and strongly enhanced near-fields for boosting both surface-driven and material-intrinsic processes^5,17,18^. Among different BIC-driven concepts^19^, metasurfaces with broken in-plane inversion symmetry are especially appealing for tailoring light-matter coupling, because they enable straightforward control over the radiative lifetimes via geometric perturbations within the meta unit^12^.

So far, most qBIC-driven metasurface realizations have relied on modifying the in-plane geometry of the resonant elements to control the asymmetry, owing to the challenges of fabricating resonators with different heights at subwavelength distances. This limitation also constitutes a significant roadblock for applications such as holography^20–22^, generation of beams with an optical angular momentum (OAM)^23^, chirality sensing^24^, and chiral nanophotonics^25–33^, which naturally require non-planar structures to enable efficient interaction with complex polarization states of light. In particular, chiral qBICs of structures with broken in-plane mirror symmetry have recently been proposed theoretically to approach the limit of maximum optical chirality^34–38^. This limit, in the most general terms, implies that an object exclusively interacts with waves of certain helicity and remains transparent to those of the opposite helicity^39^. While obtaining maximum chirality with respect to waves incident from arbitrary directions remains a sophisticated problem^40^, the path towards maximally chiral metasurfaces operating with waves incident from specific directions is more straightforward. A strict measure of metasurface chirality as formulated in ref. ^41^ requires knowledge of the asymmetry of all reflection and transmission processes. For the assessment of optical chirality in practical realizations of nanophotonic systems, it is more convenient to focus on simpler quantities obtained when a metasurface is exposed to a fixed set of circularly polarized waves. For light transmitting metasurfaces, the optimum characteristic is the difference $\Delta T$ of co-polarized transmittances, which, in the limit of maximum chirality, reaches $\pm 1$. The design of such metasurfaces is greatly facilitated by focusing on specific eigenstates that are selectively coupled to circularly polarized light of a certain handedness and give rise to ultranarrow maximally chiral resonances. However, proof-of-concept experimental implementations remain limited to the microwave range^42^, while optical realizations faced severe restrictions associated with complex three-dimensional unit-cell designs^26,42,43^ and have so far only achieved weak resonance modulation and small transmission differences Δ*T* between both handednesses of light^44,45^.

Here, we experimentally demonstrate out-of-plane symmetry-broken qBIC metasurfaces in the red part of the visible spectrum by leveraging a novel multi-step fabrication process for arbitrary height control of the resonators. Crucially, our approach implements different resonator heights based on an additional deposition step of the dielectric material, only limited by the parameters of the respective evaporation or sputtering processes. In this work, the smallest experimentally realized height difference is 10 nm, while principally a height control down to the Angstrom level is possible using appropriate nanofabrication techniques such as atomic layer deposition (ALD). We first utilize this approach to realize height-driven qBIC resonances with tailored linewidths interacting with linearly polarized light, and then we generalize this concept to demonstrate maximally chiral qBIC metasurfaces that selectively couple to circularly polarized light depending on the structural handedness. Our results and fabrication method relax the constraints of purely planar metasurface geometries, and thus offer a nontrivial generalization to the entire metasurface concept, unlocking an additional degree of freedom and extending independent parameters for freely tuning the optical response, significantly increasing their design flexibility, and delivering previously unavailable photonic functionalities requiring multi-height geometries.

## Results

### Out-of-plane qBIC engineering

The optical properties of qBIC metasurfaces can be modeled using coupled-mode theory (CMT) describing light scattering as an interference of a direct background channel and a resonant channel underpinned by the excitation and re-radiation of a qBIC eigenstate^46^. The interaction of the eigenstate with the far field is described by the coupling parameters ${m}_{{\bf{e}}}$, which, for electromagnetic waves normally incident along the *z*-axis and polarized along unit vectors **e**, are proportional to^12^:

1 $${m}_{{\bf{e}}}\propto \mathop{\int }\limits_{V}{\bf{J}}\left({\bf{r}}\right)\cdot {\bf{e}}\,{e}^{{ikz}}\,{dV}$$

where **J**(**r**) is the displacement current within the meta unit volume *V* and *k* is the light wavenumber. The coupling coefficients ${m}_{{\bf{e}}}^{{\prime} }$ to waves incident onto the metasurface backside are expressed by similar integrals with reversed propagation direction $({e}^{{ikz}}\to {{e}}^{-{ikz}}$). The corresponding power transmission coefficients of an incident wave (polarized along unit vector **i**) into an outgoing wave (polarized along unit vector **f**) are expressed as:

2 $${T}_{{\bf{fi}}}\left(\omega \right)={{\rm{|}}{t}_{{\bf{fi}}}\left(\omega \right){\rm{|}}}^{2},{\rm{with}}\,{t}_{{\bf{fi}}}\left(\omega \right)=\tau {\delta }_{{\bf{fi}}}-\frac{{m}_{{\bf{i}}}{m}_{{\bf{f}}}^{{\prime} }}{i\left(\omega -{\omega }_{0}\right)-{\gamma }_{0}}$$

where *τ* is a coefficient of background transmission preserving the polarization (${\delta }_{{\bf{fi}}}$ is the Kronecker delta symbol), and the complex eigenfrequency $({\omega }_{0}-i{\gamma }_{0})$ contains an imaginary part with radiative and dissipative contributions: ${\gamma }_{0}={\gamma }_{{\rm{r}}}+{\gamma }_{{\rm{d}}}$.

**Fig. 1:**
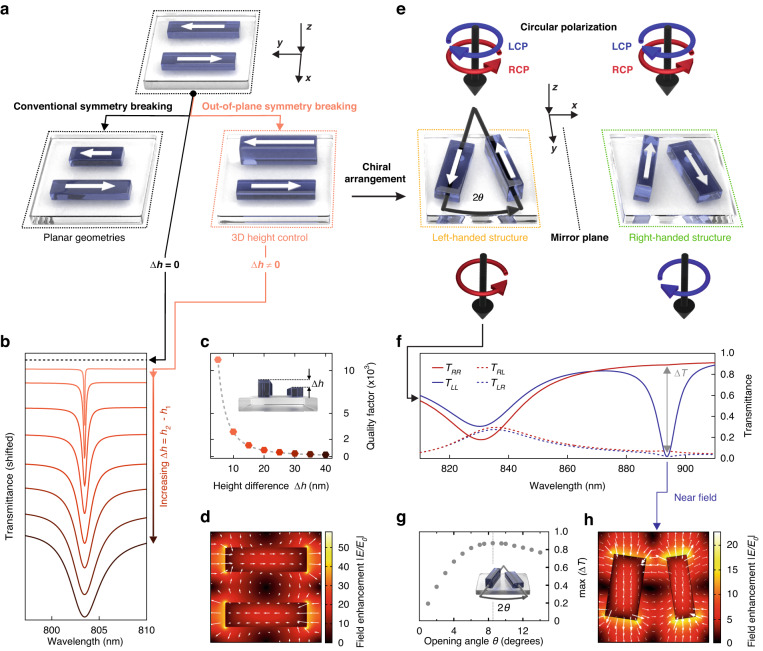
Unlocking the height of dielectric resonators for photonic qBIC engineering. **a** Established qBIC geometries use in-plane symmetry breaking to couple otherwise dark BIC states to the radiation continuum. In contrast, we introduce out-of-plane symmetry breaking for qBICs of optical metasurfaces. **b** Simulated transmittance spectra for various height differences starting from Δ*h* = 0 nm (black dashed line, top) up to Δ*h* = 40 nm (dark brown curve, bottom), with the corresponding electric near field shown in (**d**). **c** Resonance *Q* factors extracted from the spectra in (**b**) follow an inverse quadratic dependence (fitted as grey dashed line) typical for symmetry-broken qBIC metasurfaces. **e** Tailoring the height-driven qBIC by an opening angle $\theta$ creates metasurfaces with maximum chirality. **f** Spectral response of a left-handed structure for different incident circular polarizations exhibiting a pronounced qBIC resonance only for LCP light. **g** Dependence of the maximum Δ*T* on the opening angle $\theta$ showing a maximally chiral response at $\theta =8.5^\circ$. **h** Corresponding near-field of the chiral qBIC resonance

QBICs with versatile polarization properties can be realized starting from a simple symmetry-protected antiparallel electric dipole BIC of a double-rod meta unit shown in Fig. 1a. The corresponding coupling parameters given by Eq. (1) are reduced to:

3 $${m}_{{\bf{e}}}\propto {{\bf{p}}}_{1}\cdot {\bf{e}}\;{e}^{{ik}{z}_{1}}{\boldsymbol{+}}{{\bf{p}}}_{2}\cdot {\bf{e}}\,{e}^{{ik}{z}_{2}}$$

where ${{\bf{p}}}_{{1,2}}$ are the electric dipole moments of the rods 1 and 2, and ${z}_{{1,2}}$ are their effective z-coordinates. Perfect BIC isolation with ${m}_{{\bf{e}}}=0$ for all polarization unit vectors **e** is achieved when ${{\bf{p}}}_{1}=-{{\bf{p}}}_{2}$ and ${z}_{1}={z}_{2}$, i.e., when identical rods are placed within the same plane. Protection of this BIC is determined by the C_2v_ rotational symmetry of the unit cell, as the eigenstate displacement current distribution is invariant with respect to such rotation.

An exemplary in-plane perturbation that breaks the C_2v_ symmetry and transforms this BIC into a qBIC occurs when the length of one rod is varied and the rod length difference Δ*L* becomes the asymmetry parameter. For Δ*L* > 0, the electric dipole moments remain antiparallel, but have different magnitudes ($\left|{{\bf{p}}}_{1}\right|\ne \left|{{\bf{p}}}_{2}\right|$), which enables the coupling to waves linearly polarized along the long rod axis. Crucially, this coupling can also be tailored via the rod height difference $\Delta h$, which likewise breaks the C_2v_ symmetry. In this case, both the magnitudes of the dipole moments and their effective locations become slightly different. Thus, the coupling to the far field is enabled by ${m}_{y}\propto {p}_{1}({e}^{{ik}\Delta z}-1)+\Delta p$, where both $\Delta p={p}_{1}-{p}_{2}$, and ${\Delta z={z}}_{1}-{z}_{2}$ are determined by $\Delta h$ since it produces a difference in the rod volume and also shifts their centers of mass (Fig. 1b). In all above cases, the rods remain parallel and the qBIC is linearly polarized, contributing solely to ${T}_{{yy}}$.

Combining a height-driven out-of-plane symmetry breaking perturbation with a conventional in-plane one enables the realization of chiral qBIC metasurfaces^35,42^. Specifically, we implement a metasurface that breaks all point symmetries by utilizing a meta unit composed of two rods of equal lengths rotated in-plane by a small angle $\theta$ and having different footprints and heights while maintaining identical cross sections (Fig. 1e). In this case, the electric dipoles ${{\bf{p}}}_{1}$ and ${{\bf{p}}}_{2}$, even though equal in magnitude (${{\bf{|p}}}_{1}|={{\bf{|p}}}_{2}|$), are not exactly antiparallel, and their effective z-coordinates also differ, producing a three-dimensional chiral arrangement. It is insightful to evaluate Eq. (3) in such a case and obtain the coupling parameters to the left circularly polarized (LCP) and right circularly polarized (RCP) waves:

4 $${m}_{L,R}\propto \sin \left(\theta \pm k\Delta z/2\right)$$

Precise tailoring of the meta unit geometry to balance the perturbations via $2\theta =k\Delta z$ enables efficient and controllable qBIC coupling to the LCP waves with ${m}_{L}\propto \sin (2\theta )$, whereas full qBIC isolation from the RCP waves is achieved by ${m}_{R}=0$. Notably, according to Eq. (2), an ideally matched chiral metasurface does not convert circular polarizations from LCP to RCP (${t}_{{RL}}=0$) or from RCP to LCP (${t}_{{LR}}=0$), although there are no symmetry restrictions which, for example, forbid such conversions in the presence of rotational symmetry axes^47^.

Remarkably, this simple design allows approaching the ultimate limit of maximum chirality^39^, where an object remains transparent to the waves of one circular polarization, for instance RCP, and strongly interacts with those of the opposite polarization (LCP). To realize this condition, one has to first ensure that the qBIC resonance occurs in the spectral range of full background transparency ($\left|\tau \right|\approx 1$) and negligible dissipation in the metasurface material (${\gamma }_{0}={\gamma }_{{\rm{r}}}$). Then, the coupling coefficients to LCP light incident onto the metasurface from the front and the back satisfy$\,{m}_{L}^{{\prime} }=-{m}_{L}^{* }$ and the decay rate ${\gamma }_{r}$ follows a characteristic quadratic dependence on the coupling parameter ${\gamma }_{{\rm{r}}}={|{m}_{L}|}^{2}$, leading to zero transmittance ${t}_{{LL}}$ at resonance^42^ (${\omega =\omega }_{0}$) as given by Eq. (2).

Note that maximally chiral metasurfaces qualitatively outperform those exhibiting bands of similarly strong CD, such as plasmonic chiral hole arrays^48^. For the latter, the CD reaches its extreme $\pm 1$ values when one circular polarization is fully blocked regardless of the transmission of the opposite one. Strong CD of maximally chiral qBIC metasurfaces, on the contrary, is achieved when a selective blocking of waves of one circular polarization is accompanied by close to unitary transmission of their counterparts. As a specific parameter quantifying such exceptional transmission selectivity, we introduce the transmittance difference:

5 $$\Delta T={T}_{{RR}}-{T}_{{LL}}$$

which specifically characterizes the proximity of the optical response to maximum chirality. While the conventional ${CD}=({T}_{{RR}}-{T}_{{LL}})/({T}_{{RR}}+{T}_{{LL}})$ tends to $\pm 1$ as soon as waves of a certain circular polarization are fully blocked, the difference $\Delta T$ approaches $\pm 1$ only if, additionally, the waves of the opposite polarization are fully transmitted. In Fig. S1, the difference in the calculation of CD and Δ*T* is graphically represented, revealing how low transmittance of the blocked circular polarization determines CD, while the actual chiroptical response characterized by Δ*T* can remain moderate. We also note that due to the Lorentz’s reciprocity theorem, the co-polarized transmittances entering Eq. (5) do not depend on the side from which a metasurface is illuminated. This makes Eq. (5) a unique measure of transmission chirality, vanishing, for instance, for geometrically achiral metasurfaces exhibiting cross-polarized transmission asymmetry^49,50^.

Left-handed and right-handed enantiomers of the chiral qBIC metasurface can be realized by swapping the rods (Fig. 1e). The opposite enantiomer similarly remains transparent to LCP waves and resonantly blocks RCP waves. As an importantly aspect for applications and in contrast to metasurfaces with rotational symmetry axes^51,52^, the blocked circularly polarized light is not absorbed but is rather reflected backwards (Fig. S2). Therefore, under perfect circumstances, each metasurface enantiomer acts as a maximally chiral lossless filter of the corresponding handedness.

Although the above discussion is formulated in terms of electric effects, it can easily be applied to the electric-magnetic terms traditionally used to describe optical chirality. In particular, a pair of oscillating equal and antiparallel electric dipoles, shown in Fig. 1a, can also be interpreted as contributing to a magnetic dipole moment oscillating along the z-axis, which remains uncoupled from all normally incident waves in accordance with the BIC character of the state. Introducing a height difference effectively tilts the magnetic moment towards the x-axis, transforming the BIC into a qBIC that can couple to linearly polarized waves with magnetic field along the x-axis and electric field along the y-axis. Introducing a small opening angle $\theta$ only weakly affects the magnetic moment which remains approximately in the x-direction, but introduces an uncompensated electric dipole moment pointing in the same x-direction. Such pairs of collinear electric and magnetic moments are known to characterize objects exhibiting maximum optical chirality^39^. Nevertheless, since the metasurface unit cell size approaches the light wavelength, discussing its response in terms of point electric or magnetic moments is mainly used for obtaining qualitative guidance. To choose the optimal fabrication parameters and to quantitatively predict the outcome of optical experiments, we employ numerical solutions of Maxwell’s equations.

### Numerical simulations and optimization

To verify the height-driven qBIC engineering, we perform numerical simulations of the transmission and reflection of normally incident light as described in the Methods section. For linearly polarized qBICs, we model a square metasurface lattice of a period of 450 nm with meta units consisting of parallel Si rods with equal footprint of 330 × 100 nm^2^, a base height of 120 nm, and a variable height difference $\Delta h$. Simulated transmittance spectra for linearly polarized light along the rod axis are shown in Fig. 1b, revealing strong and sharp qBIC resonances in the red part of the visible spectrum as well as direct control over the resonance linewidth and modulation via the asymmetry parameter $\Delta h$. As expected for BIC-driven systems, the resonance is absent for the symmetric case ($\Delta h=0$ nm, black dashed line in Fig. 1b) and starts to couple to the radiation continuum for increasing asymmetry, showing the sharpest resonances with *Q* factors above 10^4^ for $\Delta h=5\,{\rm{nm}}$. To quantify the influence of the asymmetry parameter on the resonance sharpness, we extract the resonance *Q* factors from the transmittance spectra by fitting them with a CMT model and plotting them as a function of $\Delta h$ (Fig. 1c). We find that the *Q* factors follow an inverse quadratic relationship with the asymmetry ($Q\propto 1/{\Delta h}^{2}$), which is a hallmark feature of qBIC metasurfaces, confirming that our height-driven symmetry breaking approach fits within the established BIC framework. As a further confirmation, we simulate the electric near-field distribution at the resonant wavelength (Fig. 1d). The characteristic mode structure of antiparallel dipoles is nicely reproduced in the numerical simulations, and we observe high near-field enhancements ${|E}/{E}_{0}|$ exceeding 50, which is competitive with previous symmetry-breaking approaches^9^. Notably, the Angstrom-level control over the asymmetry provided by state-of-the-art material deposition technologies such as ALD can enable ultrasmall values of $\Delta h$ and therefore much higher field enhancements (Fig. S3). Combined with the strong confinement of fields to the resonator surface, this makes such height-driven geometries ideal candidates for enhancing interface-driven processes such as biospectroscopy or catalysis.

Next, we simulate chiral metasurface configurations by utilizing a meta unit consisting of a pair of rectangular Si rods rotated by a small angle $\theta$ away from the y-axis (Fig. 1e). The square lattice period is set to 550 nm and the rod centers are placed equidistantly with a 275 nm spacing between them. To preserve the equality of the electric dipole moment magnitudes ($\left|{{\bf{p}}}_{1}\right|={\rm{|}}{{\bf{p}}}_{2}{\rm{|}}$) we maintain an identical 160 × 100 nm^2^ cross section for both rods and keep their lengths equal to $L=310\,{\rm{nm}}$, but introduce the asymmetry by turning one rod over to its side, as shown in Fig. 1e. For the resulting $\Delta h=60\,{\rm{nm}}$, one can roughly estimate the relative displacement of the dipole moments along the z-axis by $\Delta z\approx 30\,{\rm{nm}}$. For a resonance wavelength of around 900 nm, the proportionality $2\theta =k\Delta z$ is then fulfilled for an opening angle $\theta \approx 6^\circ$. For a more precise determination, we perform a series of numerical simulations for different $\theta$ (see Fig. S4) and obtain that $\theta =8.5^\circ$ corresponds to the optimal transmission of circularly polarized waves shown in Fig. 1f. Notably, smaller angles produce weaker chirality, while larger ones also suppress the maximum values of $\Delta T$ (Fig. 1g).

In the optimal chiral configuration, as envisioned by the CMT phenomenology, the qBIC is fully decoupled from the normally incident RCP waves but gives rise to a pronounced transmission resonance for the LCP waves at a wavelength of 892 nm. The corresponding near-field pattern under LCP illumination demonstrates a maximum enhancement of the local fields by a factor of more than 20 (Fig. 1f).

### Experimental realization of height-driven BICs

**Fig. 2:**
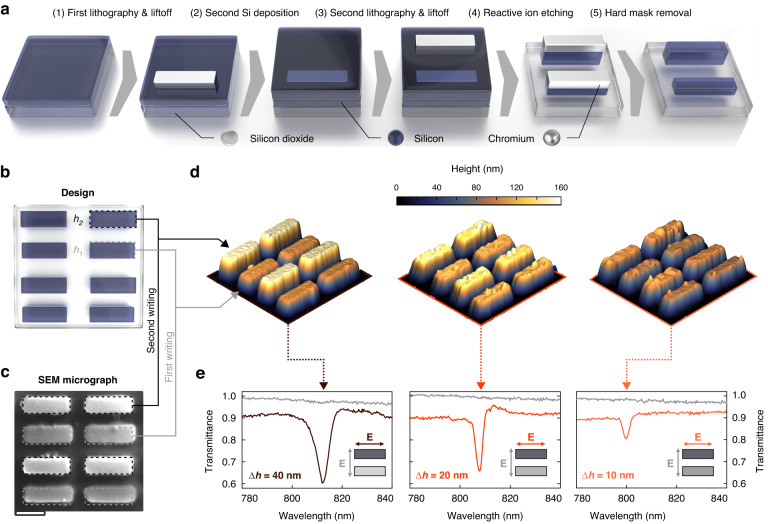
Multi-height metasurface demonstration. **a** Workflow for the fabrication of height-driven metasurfaces. **b** Schematic illustration of the two-step lithography process. **c** SEM micrograph of the fabricated multi-height metasurface (scale bar: 200 nm), where the height difference Δ*h* is already apparent from the different scattering intensities. **d** 3D AFM micrographs highlight the exact reproduction of the height differences. **e** Optical white light transmittance spectra confirm the precise control over the *Q* factor as a function of the asymmetry Δ*h*. The qBIC resonance is absent for linear polarization of the incident light along the short rod axis (grey curves in panel)

For the realization of all-dielectric metasurfaces with meta units incorporating resonators of different heights, we demonstrate a new multi-step nanofabrication approach. The core mechanism of this method leverages the combination of an *N*-step electron beam lithography process and an *N*-step deposition process to obtain a metasurface with *N* different height levels. The fabrication steps are illustrated in Fig. 2a for *N* = *2*, as required for our two-level height-driven BIC geometries. Detailed process parameters are given in the Methods. Importantly, our method is fully scalable for large *N*, since additional lithography/deposition steps can be added at any point during sample fabrication to obtain additional height levels.

Multi-height metasurface fabrication starts with plasma-enhanced chemical vapor deposition (PECVD) of an amorphous silicon (a-Si) layer onto a silicon dioxide (SiO_2_) substrate, where the thickness *h*_1_ of the layer defines the height of the resonator element with lowest thickness in the meta unit (Fig. 2a, left). Electron-beam lithography, metal deposition, and wet-chemical lift-off are then performed to obtain a thin chromium (Cr) hard mask defining the footprint of the first resonator (Fig. 2a, step (1)). Subsequently, a second layer of a-Si is deposited onto the sample with a precisely controlled thickness Δ*h*, which produces a total thickness of *h*_2_ = *h*_1_ + Δ*h* for the second resonator element (Fig. 2a, step (2)). A second hard-masking step with accurate spatial alignment is then performed to define the footprint of the second resonator (Fig. 2a, step (3)). At this point, additional pairs of a-Si and hard-masking could be performed to increase the number of height levels of the metasurface. Finally, reactive ion etching is used to transfer the resonator patterns into the a-Si layers (Fig. 2a, step (4)), resulting in pure silicon structures with different height levels after wet-chemical removal of the Cr hard masks (Fig. 2a, step (5)).

We confirm the successful fabrication of the multi-height metasurface structures by atomic force microscopy (AFM) and scanning electron microscopy (SEM). The defined metasurface pattern and height differences (Fig. 2b) are apparent from the different scattering intensities in the SEM micrograph in Fig. 2c. The SEM image further confirms the good spatial alignment between the lithography steps associated with the two resonator heights (dashed boxes in Fig. 2c and Fig. S5). We estimate our lateral alignment accuracy to be on the order of 5 nm, which is shown to have only a small impact on the optical performance in numerical simulations (Fig. S6). The AFM measurements reveal accurately defined height differences between adjacent resonators for three different height-driven metasurfaces with asymmetries of 10 nm, 20 nm, and 40 nm (Fig. 2d).

The asymmetry-dependent optical response of the metasurfaces is characterized using confocal white light transmission microscopy (Fig. 2e). As expected from our simulations, we observe pronounced qBIC resonances when the incident light is polarized along the long rod axis, whereas the resonances disappear for the orthogonal polarization consistent with the BIC mechanism. Comparing the height-driven metasurface samples for different values of Δ*h*, we find a clear increase of the resonance $Q$ factor for decreasing asymmetry, highlighting the resonance tailoring capabilities of the method. For the smallest height difference of 10 nm, the resonance modulation decreases, as the corresponding transmission feature becomes narrower but more subtle. There are several mechanisms that can possibly limit the observable Q factors, such as the finite metasurface field size, the dissipation loss in the meta-atom material as well as the scattering loss caused by small geometric deviations of the meta-atom surface and shape^4,53,54^. From a technological viewpoint, the latter factor can be suppressed by employing more precise deposition techniques, such as atomic layer deposition, and by using sapphire substrates with less surface roughness.

From our experimental results, we conclude that our fabrication approach provides an effective toolkit for realizing multi-element metasurfaces with tailored heights limited only by the precision of the utilized deposition tool. The observed transmission resonances indicate that the current PECVD technique is ideally suited to produce qBIC structures with the height difference of 20 nm and larger. Therefore, for an unambiguous demonstration of chiral qBICs, we set the height difference to 60 nm to exclude all potential fabrication-related perturbations.

### Height-driven chiral qBICs

**Fig. 3:**
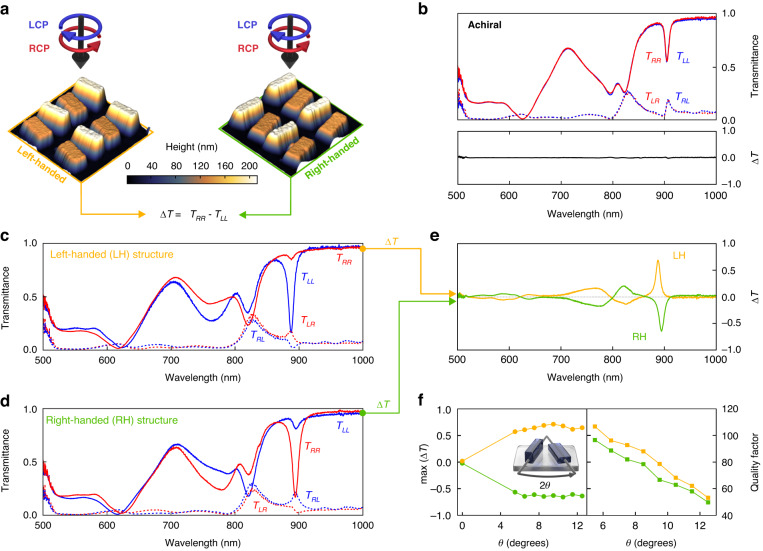
3D all-dielectric metasurfaces for chiral and spectrally sharp resonances. **a** AFM images for left-handed (yellow) and right-handed (green) structures. Both metasurfaces are illuminated with RCP and LCP light and their polarized transmittance coefficients are recorded from which we deduce the chiral transmittance difference Δ*T* = *T*_*RR*_ - *T*_*LL*_. **b** LCP and RCP transmittance spectra for the achiral metasurface showing almost identical responses in the upper panel. Lower panel: Corresponding Δ*T* spectrum showing no chiral selective response. Spectra of LCP/RCP co- and cross-polarized transmittance of the left-handed structure in (**c**) and right-handed structure in (**d**) indicate strongly selective interaction with LCP (**c**) or RCP (**d**) light. **e** Δ*T* spectra for both handednesses show the inversion of the chiral qBIC. **f** Δ*T* peak modulation for different rotation angles $\theta$ between the adjacent resonators for left-handed (yellow) and right-handed (green) structures showing a saturation in the left panel while the extracted *Q* factors decrease towards higher rotation angles as indicated in the right panel

Using our multi-step fabrication approach, we realized left-handed, right-handed, and achiral qBIC samples at a design wavelength of 900 nm approaching the visible wavelength range. Whereas the chiral metasurface realizations exhibit an opening angle $\theta$ between the two rods with different heights, the rods in the achiral metasurface remain parallel to each other (see Fig. S7 and Fig. S8 for additional AFM images and SEM images at different magnifications). The AFM images (Fig. 3a and Fig. S7) highlight the excellent experimental reproduction of the designs with clearly defined structure sizes and height differences (see Fig. S7 and Fig. S8 for more AFM images and SEM images at different magnifications). The optical response of the metasurfaces is retrieved using a home-built transmission microscopy setup incorporating the necessary polarizers and quarter wave plate to generate circularly polarized light (Fig. S9). Notably, two beam paths for RCP and LCP light are implemented, enabling the convenient switching between left-handed and right-handed illumination during experiments (for details see Methods). Light is condensed on the sample using a 10x objective and collected through an analyzer using a 60x objective to enable the polarization resolved interrogation of individual metasurface patterns with sizes of 40 by 40 µm^2^. An optical spectrometer including a grating with 150 grooves/mm is used to resolve the sharp features of the chiral qBIC resonances.

Focusing first on the achiral structures, we observe nearly identical transmission spectra for RCP and LCP illumination, leading to a vanishing Δ*T* signal (Fig. 3b, for AFM images see Fig. S10) throughout a large spectral range from 500 nm to 1000 nm. The spectral features at 900 nm in both the RCP and LCP spectra are attributed to a height-driven BIC.

As apparent from Fig. 3c, the chiral left-handed geometry shows a markedly different optical response, with a pronounced transmission dip at the design wavelength of 900 nm for LCP, which is almost completely absent for RCP illumination in line with our numerical predictions (Fig. S11). This distinct chiral selective behavior results in a large modulation of the Δ*T* signal with Δ*T* = 0.7 and a narrow bandwidth with a quality factor of *Q* ≈ 80, which is, to the best of our knowledge, the highest experimental value for a maximally chiral optical resonance reported in the literature so far. For the chiral right-handed structure in Fig. 3d, the situation is reversed, with a strong resonance feature for RCP light and a vanishing optical response for LCP, resulting in a Δ*T* value with equally large magnitude but opposite sign at Δ*T* = −0.7, and a similarly high $Q$ factor as obvious from Fig. 3e, f (for full spectra see Figs. S12, S13). Most importantly, the cross-polarization measurements in Fig. 3d show no significant polarization conversion as expected from Eq. (2) and our numerical predictions (Fig. 1f). We also simulated the distribution of optical chirality density (OCD) for the achiral and chiral left-handed metasurface (Fig. S14), showing strong OCD values for the LH metasurface upon illumination with LCP illumination, while contrarily almost no OCD occurs for RCP illumination.

Additionally, the chiral qBIC retains the versatile resonance tuning capabilities of the BIC concept, which we demonstrate by experimentally varying the opening angle $\theta$ between the two rods. We find that the modulation of the Δ*T* signal (Fig. 3f, left-hand side) is mostly constant while the *Q* factor of the resonance (Fig. 3f, right-hand side) can be tailored via the opening angle for $\theta \ge 5^\circ$. Specifically, lower values of $\theta$ produce higher $Q$ factors, which can be beneficial for spectrally selective chiral applications, whereas the signal modulation vanishes when approaching the achiral case ($\theta =0^\circ$). Here, the BIC-based resonance tuning can be harnessed to precisely configure the optical system for the target chiral use case, striking a balance between $Q$ factor and modulation as required.

Furthermore, to estimate the accuracy of optical experiments and to check extrinsic 3D chirality effects due to symmetry-breaking with the substrate, we examine the consequences of Lorentz reciprocity by performing measurements on the chiral metasurface when excited from the top and the bottom. Since the spectral responses remain unchanged (Fig. S15) we conclude that our optical setup is sufficiently precise, as also confirmed by our in-depth analysis of the optical response as a function of sample rotation (Fig. S16).

## Discussion

Our experimental polarization-dependent transmittance spectra clearly identify the observed resonance at a wavelength of around 900 nm as a chiral qBIC closely tuned to the maximum chirality regime, with the transmittance difference reaching $\Delta T=\pm 0.7$. Thus, for the right-handed (left-handed) enantiomer, the co-polarized LCP (RCP) transmittance stays close to 0.9, while the co-polarized RCP (LCP) transmittance resonantly drops down to below 0.2, while both cross-polarized transmittances remain below 0.1 within the whole range of this resonance.

Qualitatively, regarding the selective RCP/LCP transmission, the qBIC metasurface represents a maximally chiral version of previous intrinsically chiral and rotationally symmetric metasurfaces^55,56^ or, in even simpler terms, a maximally chiral analog of a bi-isotropic Pasteur medium^57^. However, while the previous examples absorb the attenuated circularly polarized waves, here, on the contrary, the qBIC metasurface reflects them with up to 80 % efficiency (see Fig. S2). This peculiar type of near-lossless maximum chirality has been previously observed in model microwave experiments^42^, and we present its first optical realization.

Until now, selective reflection of circularly polarized light was attributed to the so-called chiral mirrors, which, however, inevitably invert the circular polarization of the transmitted light^58^. Recently, such mirrors have been experimentally realized as perforated silicon slabs^49^ and as arrays of silicon particles of reduced symmetry hosting specific qBICs^26^. Being achiral, as all planar metasurfaces, such structures exhibit intriguing optical performance, which, however, emphasizes the limits of the planar design and fabrication. Unlocking the out-of-plane dimension enables creating optical structures hosting strongly chiral qBICs selectively coupled to light of a specific helicity. Even though our height-driven chiral metastructures involve only a single layer of carefully placed rectangular blocks upon a flat substrate, they achieve much stronger and, importantly, much more controllable optical chirality compared to sophisticated double-layer arrangements of metal^59^ or silicon^57^ blocks. On a larger scale, analogous resonators can be arranged as Fabry-Perot cavities confined between planar chiral mirrors^50^, although the practical feasibility of such cavities is yet to be demonstrated.

In summary, we have suggested and realized a novel nanofabrication approach that allows for the implementation of multi‐height metasurfaces to achieve the precise tailoring of the *Q* factor in symmetry‐broken qBIC metasurfaces via the height difference of the meta‐unit resonators. The fabrication of such nanostructures is based on newly developed multistep lithographic techniques. This seemingly incremental increase of the fabrication complexity delivers, however, a qualitative leap in the optical design: The new degree of freedom allows for the simultaneous tailoring of the qBIC chirality and *Q* factor, thus opening vast prospects for multi‐functional and lossless metasurfaces impacting nonlinear chiral optics and lasing, chirality sensing and, prospectively, enantiomer‐selective photochemistry.

## Materials and methods

### Numerical simulations

The refractive indexes of the SiO_2_ substrate and Si rods were taken from the data of in-house white-light ellipsometry (Fig. S17). Numerical simulations of the linear height-driven BIC structures were carried out with CST Microwave Studio, determining the spectral responses of height-driven metasurfaces in the frequency domain. Light transmission and reflection by the chiral metasurfaces were numerically investigated by finite-element method (FEM) using the Electromagnetic Waves Frequency Domain module of COMSOL Multiphysics in 3D mode. The tetrahedral spatial mesh for FEM was automatically set by the COMSOL physics-controlled preset. The simulations were performed within a rectangular spatial domain containing one metasurface meta unit with periodic boundary conditions set on its sides and with the excitation and registering circularly polarized ports at the top and the bottom. In Fig. S11, for the wavelength range below the diffraction cutoff, appropriate registering diffraction ports were added. To assess the simulation precision, the transmission and reflection coefficients bound by Lorentz reciprocity were independently obtained and compared, which ensured that the evaluated values are correct with a 1% accuracy. For reproducing experimental spectra, the dimensions of the structures were adjusted to the acquired AFM and SEM data.

### Nanostructure fabrication

Prior to the fabrication, the silicon dioxide (SiO_2_) substrates were cleaned via sonication in acetone at 55 °C, rinsed in isopropanol (IPA), and dried under nitrogen (N_2_) flux, followed by oxygen (O_2_) plasma etching to remove organic residues. After the cleaning process, 100 nm of amorphous silicon (a-Si) were deposited onto the SiO_2_ substrates via plasma-enhanced chemical vapor deposition (PECVD) using a silane (SiH_4_) precursor gas at a substrate temperature of 250 °C via a PlasmaPro 100 PECVD from Oxford Instruments. The height-driven all-dielectric metasurface fabrication was based on a three-step electron beam lithography (EBL) process. In particular, all EBL processes were performed using an eLINE Plus from Raith Nanofabrication with an acceleration voltage of 30 kV and a 15 µm aperture by patterning a double layer of polymethylmethacrylate (PMMA), a positive-tone electron beam resist, obtained via spin-coating and a subsequent soft-bake step (80 nm of PMMA 950k A2 on top of 100 nm of PMMA 495k A4 from Kayaku Advanced Materials). Subsequent development in a 3:1 IPA:MIBK (methyl isobutyl ketone) solution followed by the electron beam hard mask evaporation and an overnight liftoff using a PMMA remover (Microposit remover 1165) transferred the exposed pattern into the desired nanostructures.

In the first patterning process, a 100 nm thick gold marker system was defined on top of the 100 nm thin a-Si film, which was used for aligning the following two fabrication steps of the metasurfaces with different heights. This marker system enabled a spatial control of the relative nanostructure position between the two fabrication steps of $\le$ 5 nm. In the second patterning run, only the lower elements of the meta units were written and developed followed by the deposition of a 25 nm chromium (Cr) on 20 nm SiO_2_ hard mask. Subsequent deposition of an additional a-Si layer (here 10 nm, 20 nm, and 40 nm) homogeneously covered the sample and buried all structures beneath. The third lithography step was used to define the taller elements within the meta unit, again with a 25 nm Cr mask on top of 25 nm SiO_2_.

Finally, the hard mask pattern was transferred into the silicon via reactive ion etching (RIE) using a PlasmaPro 100 ICP-RIE from Oxford Instruments in a mixture of 7 sccm Ar and 20 sccm Cl_2_ at 20 W bias power and 200 W induction power. After etching, the chromium hard mask was removed in a wet etchant to retrieve the silicon structures. The SiO_2_ capping layer was removed in another RIE step using 30 sccm Ar and 20 sccm CHF_3_ under 30 W bias power and the sample was cleaned in gentle oxygen plasma before the optical measurements.

### Optical characterization of height-driven qBICs

The optical characterization of the achiral height-driven qBIC metasurfaces was performed in a commercially available white light confocal microscopy setup (Witec alpha 300 series). The sample was illuminated with linearly polarized and collimated white light from the bottom by a broadband halogen lamp (Thorlabs OSL2). Afterwards, the transmitted signal was collected with a 10x (NA = 0.25) objective and confocally detected by coupling it into a multimode fiber. From there, the light was guided into the spectrometer where it impinged onto a reflective grating (150 grooves/mm) and was dispersed onto a silicon CCD. For each spectrum, the transmitted intensity through the qBIC metasurface was normalized to the bare SiO_2_ substrate to retrieve the transmittance spectrum and remove any unwanted features from the optical beam path. For the acquisition of an individual spectrum, 40 accumulations were taken at 0.5 s each.

### Optical characterization of the chiral qBICs

Optical characterization was performed using a custom-built transmission microscope. A schematic overview over the set-up is given in Fig. S9a. The setup is driven by the collimated output of a fiber-coupled supercontinuum white light laser (SuperK FIANIUM from NKT Photonics) set to a power of 5% of the maximum value and a repetition rate of 0.302 MHz. The beam was guided to a polarizing beam splitter (PBS), where the beam is split in two paths, generating horizontal (HP) or vertical (VP) linear polarization (2x LPVIS100 from ThorLabs, 550–1500 nm), respectively. Together with the quarter wave plate (QWP, RAC4.4.20 from B-Halle, 500–900 nm), circularly polarized light (CPL) was generated. The polarization could be varied between RCP and LCP by blocking one of the two beam paths generating HP or VP. This eliminates the need to rotate the polarizers or QWP, which can induce elliptical polarization if not controlled after rotation. The QWP was located directly below the objectives to avoid any reflections off mirrors, which can turn CPL into elliptically polarized light while linearly polarized light remains unaffected.

Light was condensed on the sample using a 10x objective (Olympus PLN, NA = 0.25) and collected using a 60x objective (Nikon MRH08630, NA = 0.7). After alignment, the beam was slightly defocused to illuminate the entire metasurface area of 40 µm x 40 µm (Fig. S9b). The desired location for the measurement was selected by using an aperture after the objectives. For the measurement of the co- and cross-polarization terms, a chiral analyzer, consisting of a QWP (AQWP05-580 from Thorlabs, 350–850 nm) and a linear polarizer (WP25M-UB from Thorlabs, 250–4000 nm), were installed after the collection objective. Using a flip mirror, the light was then guided directly to the CCD camera or to the spectrometer by using a multimode fiber (Thorlabs M15L05, core size: 105 µm, NA = 0.22). A spectrometer from Princeton Instruments was used with a grating period of 300 g/mm, blaze angle of 750 nm and spectral resolution of 0.13 nm. All spectra were recorded using a binning of one line, an exposure duration of 50 ms and 50 spectra were accumulated. Prior to measurement, the dark noise was recorded by blocking the illumination. All spectra were referenced by using a background measurement, which was performed on the same substrate far away from the metasurfaces.

### Supplementary information

Supplementary Information

## Data Availability

The main data supporting the findings of this study are available within the article and its Supplementary Information files. Extra data are available from the corresponding author upon reasonable request.

## References

[1] Hu YQ (2020). All-dielectric metasurfaces for polarization manipulation: principles and emerging applications. Nanophotonics.

[2] Metalenz PolarEyes. at https://www.metalenz.com/products/polareyes-polarization-sensing-module/.

[3] Cortés E (2022). Optical metasurfaces for energy conversion. Chem. Rev..

[4] Liu ZJ (2019). High-*Q* quasibound states in the continuum for nonlinear metasurfaces. Phys. Rev. Lett..

[5] Bernhardt N (2020). Quasi-BIC resonant enhancement of second-harmonic generation in WS_2_ monolayers. Nano Lett..

[6] Lin DM (2014). Dielectric gradient metasurface optical elements. Science.

[7] Yu NF, Capasso F (2014). Flat optics with designer metasurfaces. Nat. Mater..

[8] Jahani Y (2021). Imaging-based spectrometer-less optofluidic biosensors based on dielectric metasurfaces for detecting extracellular vesicles. Nat. Commun..

[9] Tittl A (2018). Imaging-based molecular barcoding with pixelated dielectric metasurfaces. Science.

[10] Yesilkoy F (2019). Ultrasensitive hyperspectral imaging and biodetection enabled by dielectric metasurfaces. Nat. Photonics.

[11] Azzam SI, Kildishev AV (2021). Photonic bound states in the continuum: from basics to applications. Adv. Opt. Mater..

[12] Koshelev K (2018). Asymmetric metasurfaces with high-*Q* resonances governed by bound states in the continuum. Phys. Rev. Lett..

[13] Luk’yanchuk B (2010). The Fano resonance in plasmonic nanostructures and metamaterials. Nat. Mater..

[14] Koshelev K (2019). Nonradiating photonics with resonant dielectric nanostructures. Nanophotonics.

[15] Ha ST (2018). Directional lasing in resonant semiconductor nanoantenna arrays. Nat. Nanotechnol..

[16] Leitis A (2019). Angle-multiplexed all-dielectric metasurfaces for broadband molecular fingerprint retrieval. Sci. Adv..

[17] Anthur AP (2020). Continuous wave second harmonic generation enabled by quasi-bound-states in the continuum on gallium phosphide metasurfaces. Nano Lett..

[18] Koshelev K (2020). Subwavelength dielectric resonators for nonlinear nanophotonics. Science.

[19] Hsu CW (2016). Bound states in the continuum. Nat. Rev. Mater..

[20] Genevet P, Capasso F (2015). Holographic optical metasurfaces: a review of current progress. Rep. Prog. Phys..

[21] Zhao RZ, Huang LL, Wang YT (2020). Recent advances in multi-dimensional metasurfaces holographic technologies. PhotoniX.

[22] Ren HR (2020). Complex-amplitude metasurface-based orbital angular momentum holography in momentum space. Nat. Nanotechnol..

[23] Weber K (2017). Single mode fiber based delivery of OAM light by 3D direct laser writing. Opt. Express.

[24] Warning LA (2021). Nanophotonic approaches for chirality sensing. ACS Nano.

[25] Schäferling, M. Chiral Nanophotonics: Chiral Optical Properties of Plasmonic Systems. (Cham: Springer, 2017).

[26] Shi T (2022). Planar chiral metasurfaces with maximal and tunable chiroptical response driven by bound states in the continuum. Nat. Commun..

[27] Kim KH, Kim JR (2021). High-*Q* chiroptical resonances by quasi‐bound states in the continuum in dielectric metasurfaces with simultaneously broken in‐plane inversion and mirror symmetries. Adv. Opt. Mater..

[28] Zhu AY (2018). Giant intrinsic chiro-optical activity in planar dielectric nanostructures. Light.Sci. Appl..

[29] Ji CY (2021). Artificial propeller chirality and counterintuitive reversal of circular dichroism in twisted meta-molecules. Nano Lett..

[30] Hentschel M (2017). Chiral plasmonics. Sci. Adv..

[31] Wu C (2014). Spectrally selective chiral silicon metasurfaces based on infrared fano resonances. Nat. Commun..

[32] Valev VK (2013). Chirality and chiroptical effects in plasmonic nanostructures: fundamentals, recent progress, and outlook. Adv. Mater..

[33] Vinçon I (2022). Strong polarization dependent nonlinear excitation of a perovskite nanocrystal monolayer on a chiral dielectric nanoantenna array. ACS Photonics.

[34] Overvig A, Yu NF, Alù A (2021). Chiral quasi-bound states in the continuum. Phys. Rev. Lett..

[35] Gorkunov MV, Antonov AA, Kivshar YS (2020). Metasurfaces with maximum chirality empowered by bound states in the continuum. Phys. Rev. Lett..

[36] Dixon J (2021). Self-isolated raman lasing with a chiral dielectric metasurface. Phys. Rev. Lett..

[37] Wu JJ (2021). Observation of giant extrinsic chirality empowered by quasi-bound states in the continuum. Phys. Rev. Appl..

[38] Liu WZ (2019). Circularly polarized states spawning from bound states in the continuum. Phys. Rev. Lett..

[39] Fernandez-Corbaton I, Fruhnert M, Rockstuhl C (2016). Objects of maximum electromagnetic chirality. Phys. Rev. X.

[40] Garcia-Santiago X (2022). Toward maximally electromagnetically chiral scatterers at optical frequencies. ACS Photonics.

[41] Garcia-Santiago X (2017). Measuring the electromagnetic chirality of 2D arrays under normal illumination. Opt. Lett..

[42] Gorkunov MV (2021). Bound states in the continuum underpin near‐lossless maximum chirality in dielectric metasurfaces. Adv. Opt. Mater..

[43] Kupriianov AS (2019). Metasurface engineering through bound states in the continuum. Phys. Rev. Appl..

[44] Chen Y (2023). Observation of intrinsic chiral bound states in the continuum. Nature.

[45] Zhang XD (2022). Chiral emission from resonant metasurfaces. Science.

[46] Fan SH, Suh W, Joannopoulos JD (2003). Temporal coupled-mode theory for the Fano resonance in optical resonators. J. Opt. Soc. Am. A.

[47] Menzel C, Rockstuhl C, Lederer F (2010). Advanced Jones calculus for the classification of periodic metamaterials. Phys. Rev. A.

[48] Kondratov AV (2016). Extreme optical chirality of plasmonic nanohole arrays due to chiral Fano resonance. Phys. Rev. B.

[49] Semnani B (2020). Spin-preserving chiral photonic crystal mirror. Light.Sci. Appl..

[50] Voronin K (2022). Single-handedness chiral optical cavities. ACS Photonics.

[51] Kaschke, J. et al. Metamaterial circular polarizers based on metal N-helices. CLEO: 2013. San Jose: IEEE, (2013). 10.1364/OE.20.026012 23187416

[52] Gorkunov MV (2014). Extreme optical activity and circular dichroism of chiral metal hole arrays. Appl. Phys. Lett..

[53] Kühne J (2021). Fabrication robustness in BIC metasurfaces. Nanophotonics.

[54] Yang YM (2014). All-dielectric metasurface analogue of electromagnetically induced transparency. Nat. Commun..

[55] Gorkunov MV (2018). Chiral visible light metasurface patterned in monocrystalline silicon by focused ion beam. Sci. Rep..

[56] Tanaka K (2020). Chiral bilayer all-dielectric metasurfaces. ACS Nano.

[57] Lindell, I. V. et al. Electromagnetic Waves in Chiral and Bi-Isotropic Media. (Boston: Artech House Publishers, 1994).

[58] Plum E, Zheludev NI (2015). Chiral mirrors. Appl. Phys. Lett..

[59] Yin XH (2015). Active chiral plasmonics. Nano Lett..

