# Supplementary material for: Unlocking the out-of-plane dimension for photonic bound states in the continuum to achieve maximum optical chirality
Source: Light Sci Appl. 2023 Oct 12;12:250. doi: 10.1038/s41377-023-01295-z (PMC10570380; doi:10.1038/s41377-023-01295-z)
Supplement: Supplementary file 1 — Supplementary Information [file 41377_2023_1295_MOESM1_ESM.pdf]

Supporting Information for

Unlocking the out-of-plane dimension for photonic bound states in the continuum to achieve maximum optical chirality

Lucca Kühner[1,†], Fedja J. Wendisch[1,†], Alexander A. Antonov[2], Johannes Bürger[1],

Ludwig Hüttenhofer[1], Leonardo de S. Menezes[1,3], Stefan A. Maier[1,4,5], Maxim V. Gorkunov[2],

Yuri Kivshar[6,*], and Andreas Tittl[1,*]

[1]Chair in Hybrid Nanosystems, Nanoinstitute Munich, and Center for NanoScience, Faculty of Physics, Ludwig-Maximilians-University Munich, Königinstrasse 10, 80539 München, Germany

[2]Shubnikov Institute of Crystallography, FSRC “Crystallography and Photonics”, Russian Academy of Sciences, Moscow 119333, Russia

[3]Departamento de Física, Universidade Federal de Pernambuco, 50670-901 Recife, Pernambuco, Brazil

[4]School of Physics and Astronomy, Monash University, Wellington Rd, Clayton VIC 3800, Australia

[5]The Blackett Laboratory, Department of Physics, Imperial College London, London, SW7 2AZ, United Kingdom

[6]Nonlinear Physics Centre, Research School of Physics, Australian National University, Canberra ACT 2601, Australia

[†]These authors contributed equally

*e-mail: yuri.kivshar@anu.edu.au, andreas.tittl@physik.uni-muenchen.de

Contents

Figure S1. Graphical comparison between the calculation of CD (a) and ΔT (b) for different arbitrary values of T_{RR} and T_{LL}. 3

Figure S2: Simulated reflectance spectra of a left-handed chiral qBIC enantiomer. 4

Figure S3: Angstrom-level control for high field enhancements. 5

Figure S4: Simulated transmittance spectra for different opening angles $\boldsymbol{\theta}$. 6

Figure S5: Lateral resolution of the multi-step nanofabrication approach. 7

Figure S6: Influence of misalignment on the height-driven BIC spectra. 8

Figure S7: Fabrication and characterization of the achiral and chiral qBIC metasurfaces. 9

Figure S8. SEM images of the achiral and chiral qBIC metasurfaces at different magnifications. 10

Figure S9: Overview over the set-up for optical characterization of the chiral qBICs. 11

Figure S10: AFM images of the achiral qBIC metasurface. 12

Figure S11: Comparison between experiment and simulation. 13

Figure S12: All experimental spectra of the achiral and chiral qBIC metasurfaces with opening angles from 0-8.5°. 14

Figure S13: All experimental spectra of the achiral and chiral qBIC metasurfaces with opening angles from 9.5-12.5°. 15

Figure S14: Distributions of optical chiral density (OCD) for the achiral and the chiral left-handed metasurface. 16

Figure S15: Lorentz reciprocity of the chiral qBIC. 17

Figure S16: Influence of sample rotation on the measurements of the chiral qBIC metasurfaces with chiral analyzer. 18

Figure S17. Refractive index of the amorphous silicon. 19

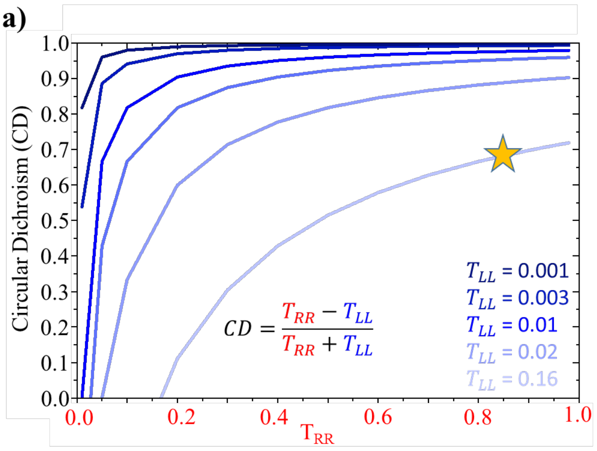

Figure S1. Graphical comparison between the calculation of CD (a) and ΔT (b) for different arbitrary values of T_{RR} and T_{LL}.

Values of T_{RR} are given on the x-axis, while values of T_{LL} are shown in the plots with different colors. For small values of the opposite polarization (here T_{LL}), the CD plot saturates and quickly reaches the extreme values of +/-1. An effective way of increasing the CD is thereby blocking the opposite polarization, rather than increasing the chiroptical response. Contrarily, ΔT always follows a linear trend, and only achieves its extreme values +/- 1, if one certain circular polarization is fully blocked, while the opposite polarization is fully transmitted, hence is more suitable to characterize maximum chirality. The yellow star indicates the values achieved for the chiral qBICs in our work.

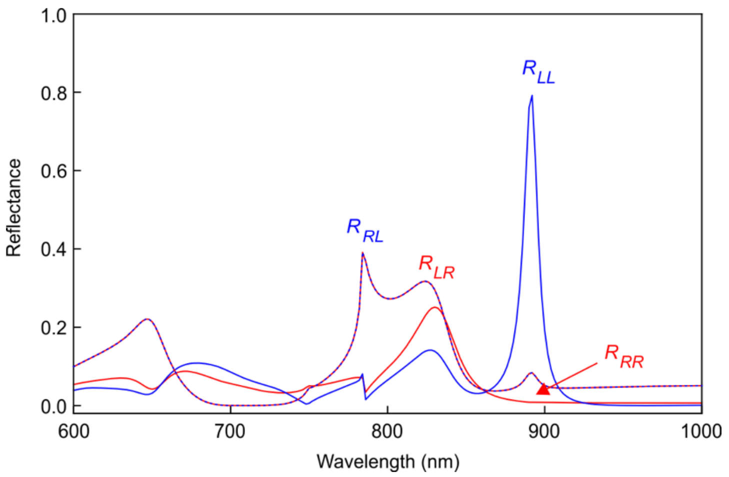

Figure S2: Simulated reflectance spectra of a left-handed chiral qBIC enantiomer.

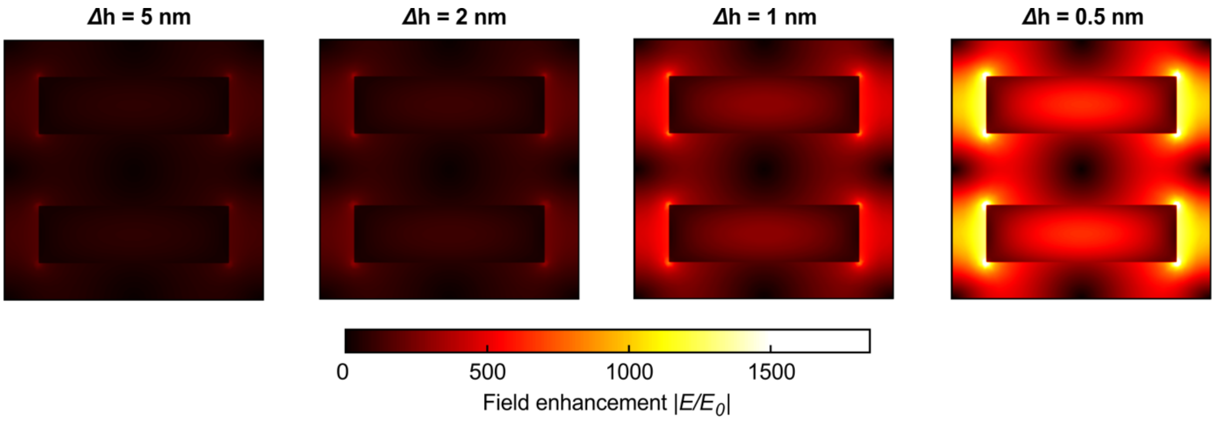

Figure S3: Angstrom-level control for high field enhancements.

Numerical investigation of the electric near-fields associated with the height-driven qBIC resonances for various small height differences. The field enhancement increases drastically towards Angstrom-scale height differences, such as 5 Å on the right-hand side with field enhancements of up to 2000.

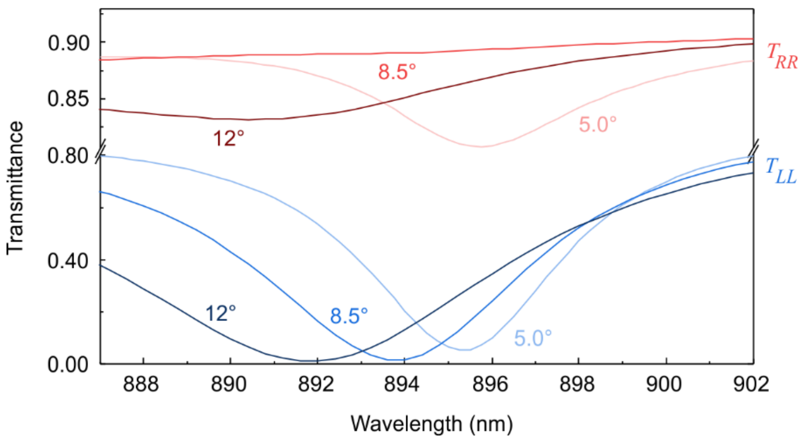

Figure S4: Simulated transmittance spectra for different opening angles θ.

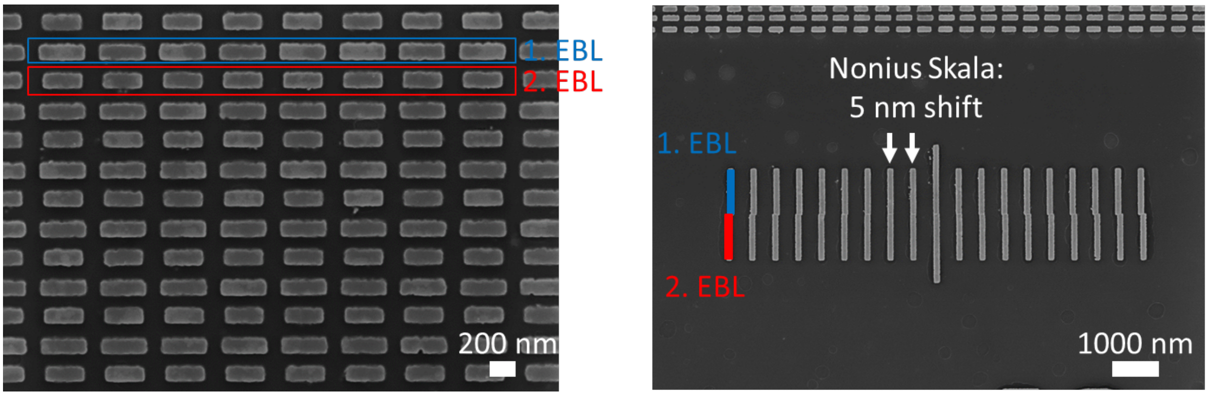

Figure S5: Lateral resolution of the multi-step nanofabrication approach.

SEM images proving the lateral resolution of the multi-step nanofabrication approach. Left: Fabricated metasurfaces with two electron beam lithography (EBL) steps, where both resonators have the same height. Right: Nonius scale for determining the shift between both EBL steps. The lines at the top and bottom have been written with two EBL steps (marked blue and red). From the center (longest line), each line is shifted by $\pm$ 5 nm for the second EBL step, revealing a spatial resolution of < 5 nm, which can be taken into account during the sample fabrication.

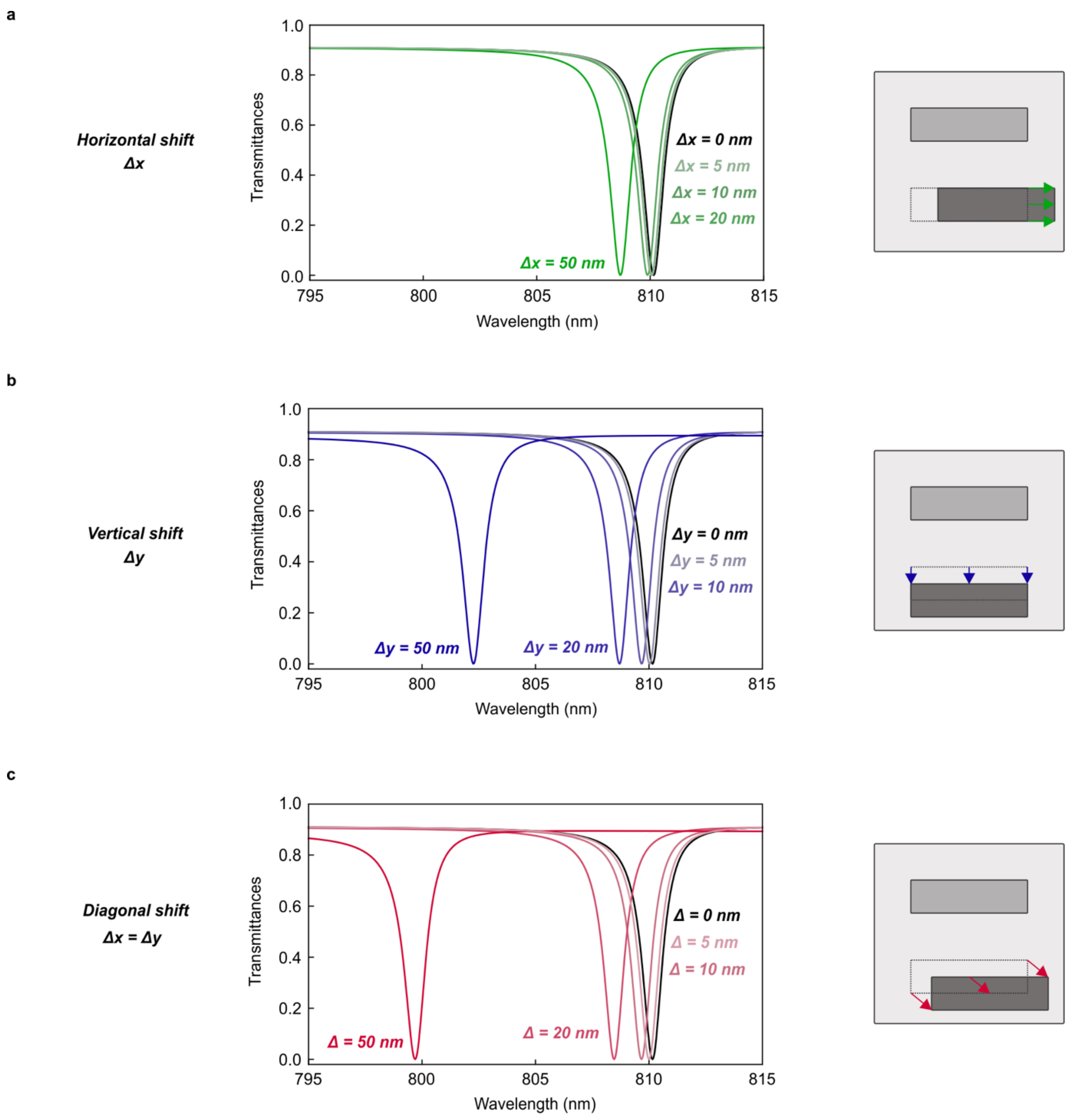

Figure S6: Influence of misalignment on the height-driven BIC spectra.

Simulated spectral responses for small shifts in x (panel a), in y (panel b), and in diagonal direction (panel c) show no significant resonance shifts for alignment accuracies below 10 nm. The properties of the resonance also remain mostly unchanged.

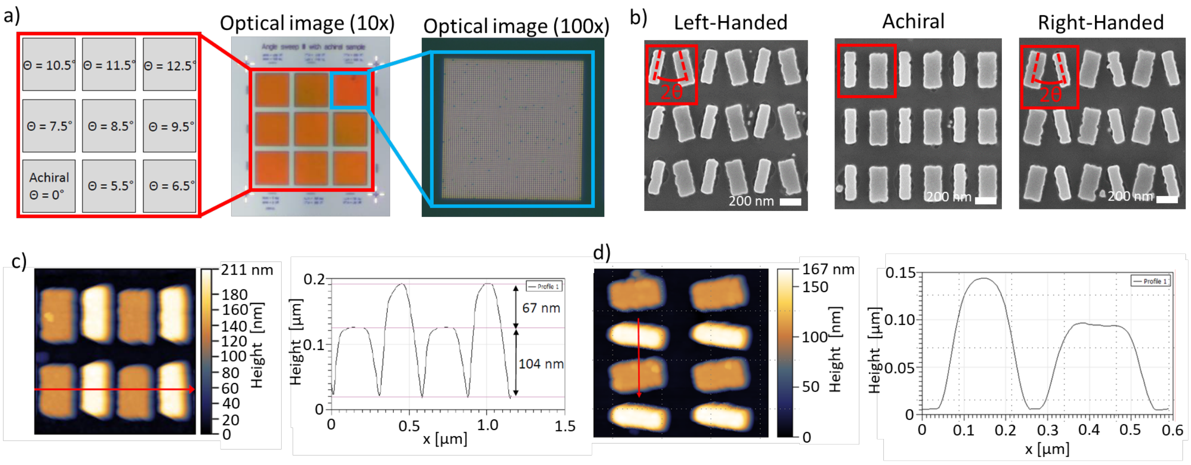

Figure S7: Fabrication and characterization of the achiral and chiral qBIC metasurfaces.

a, Left: Overview over the different achiral and chiral qBIC metasurfaces with varying opening angles. Middle: Optical image (10x) of the fabricated qBIC metasurfaces. Right: Optical image (100x) of the chiral qBIC metasurfaces with opening angle of 12.5°. **b,** SEM images of the achiral and chiral left- and right-handed sample. The meta-atom with larger height is visible by its brighter contrast. **c, d,** AFM measurements and line scans of the achiral (**c**) and chiral (**d**) qBIC metasurface.

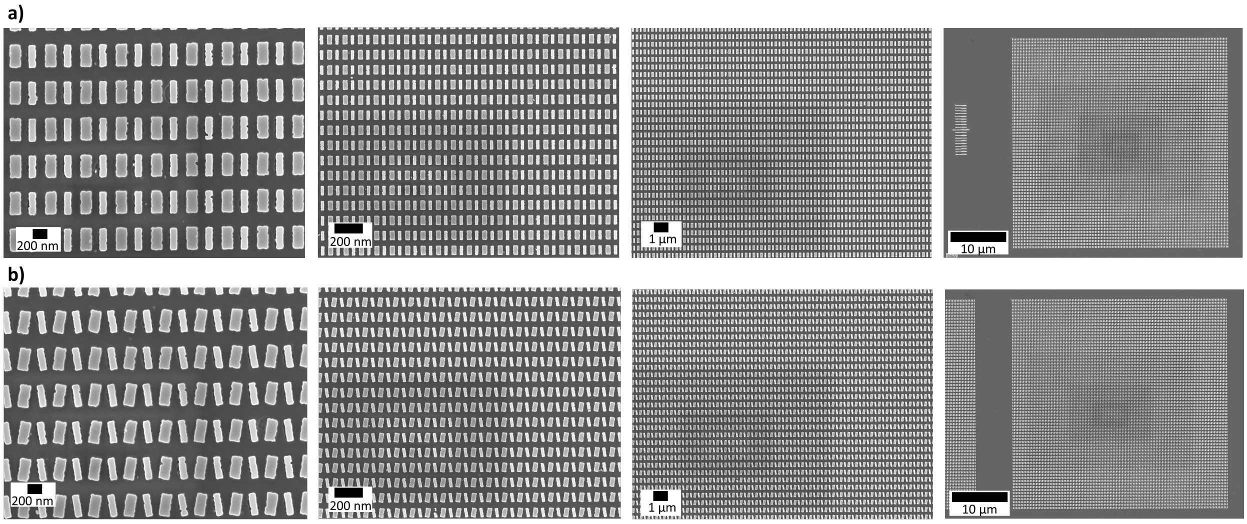

Figure S8. SEM images of the achiral and chiral qBIC metasurfaces at different magnifications.

a, Achiral metasurface with opening angle of 0°. **b,** Chiral metasurface with opening angle of 8.5°.

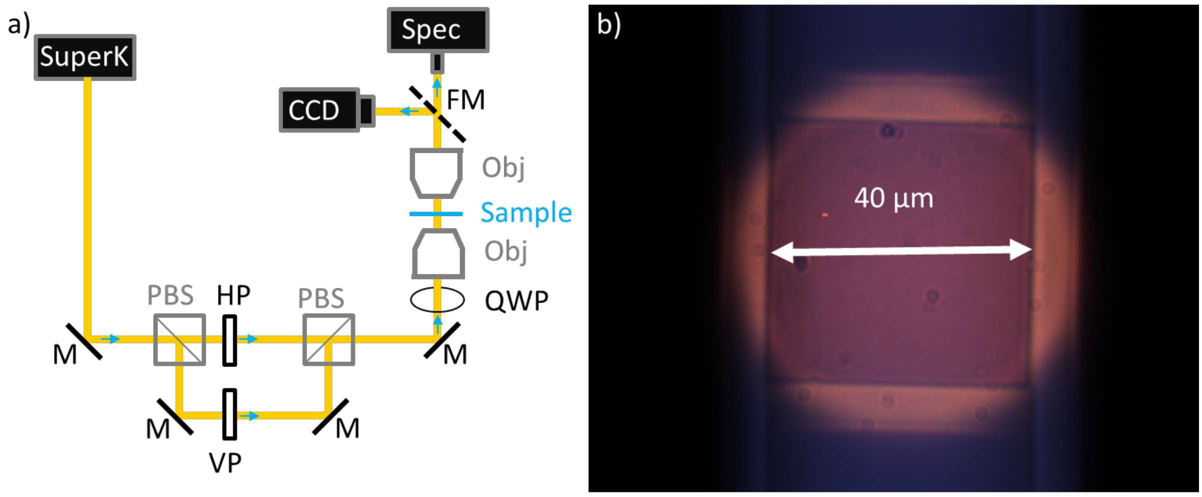

Figure S9: Overview over the set-up for optical characterization of the chiral qBICs.

a, Schematic overview of the optical characterization setup. M (solid black line): mirror, FM (dashed black line): flip mirror. PBS: polarizing beam splitter. HP: horizontal linear polarizer. VP: vertical linear polarizer. QWP: quarter wave plate. Obj.: microscope objective. To generate RCP or LCP light, either HP or VP beam path is blocked. **b,** Image of the qBIC metasurface captured on the CCD camera. For the measurements, the iris aperture was reduced to fit into the metasurface.

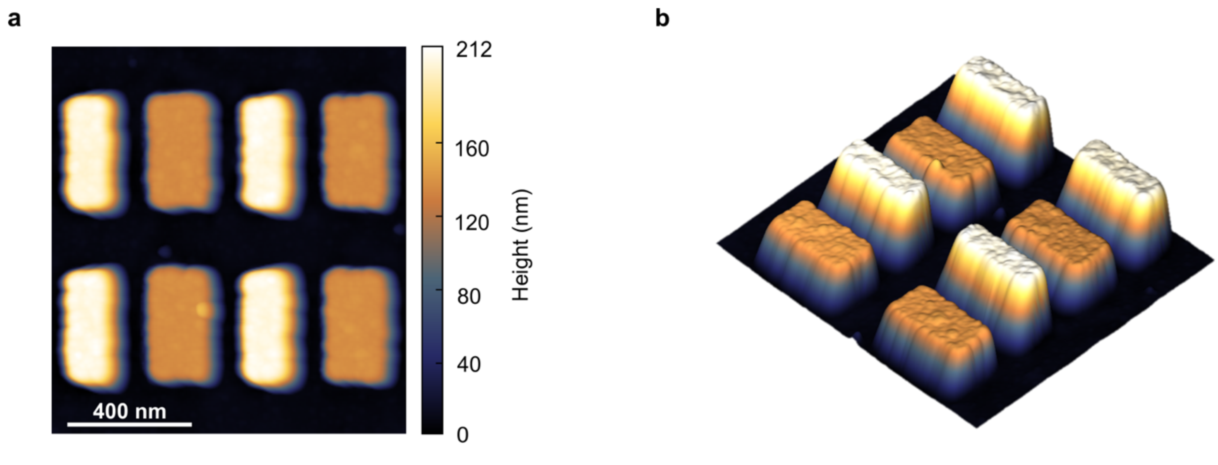

Figure S10: AFM images of the achiral qBIC metasurface.

a, Planar and **b,** 3-D AFM image for the achiral qBIC metasurface.

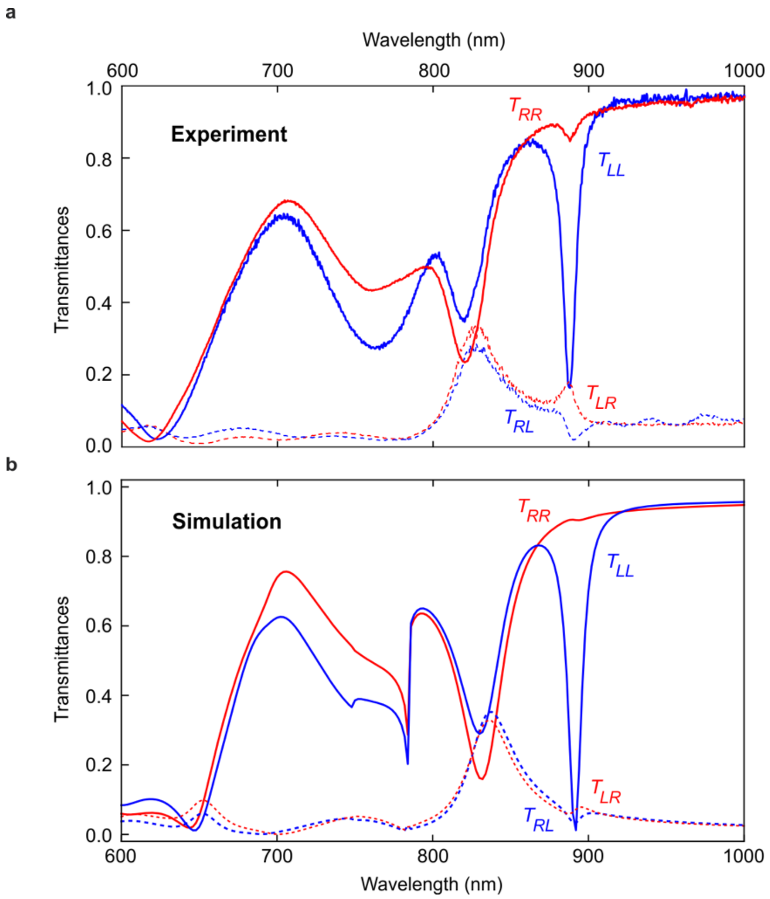

Figure S11: Comparison between experiment and simulation.

The measured chiral response (upper panel) for a left-handed structure with an opening angle of $\boldsymbol{\theta} = \mathbf{8.5}°$ only interacts with LCP light (blue curve T_{LL}) for which the chiral qBIC is excited. The experimental data show excellent agreement with our numerical simulations (lower panel). We used scanning electron microscopy (SEM) images to match the lateral dimensions, atomic force microscopy scans (see Fig. S6 for SEM and AFM data) to determine the different heights of the resonators and utilized the in-house measured amorphous silicon ellipsometry data to match the numerical simulations as precisely as possible to the measurement results. In particular, the simulations were done for periodicities of 515 nm and 540 nm in the x- and y-directions correspondingly, heights of $h_1 = 155$ nm and $h_2 = 95$ nm (see Fig. S7), an opening angle of $\theta = 8.5°$, rod widths of $w_1 = 104$ nm and $w_2 = 157$ nm, and lengths of $l_1 = \ l_2 = 334$ nm.

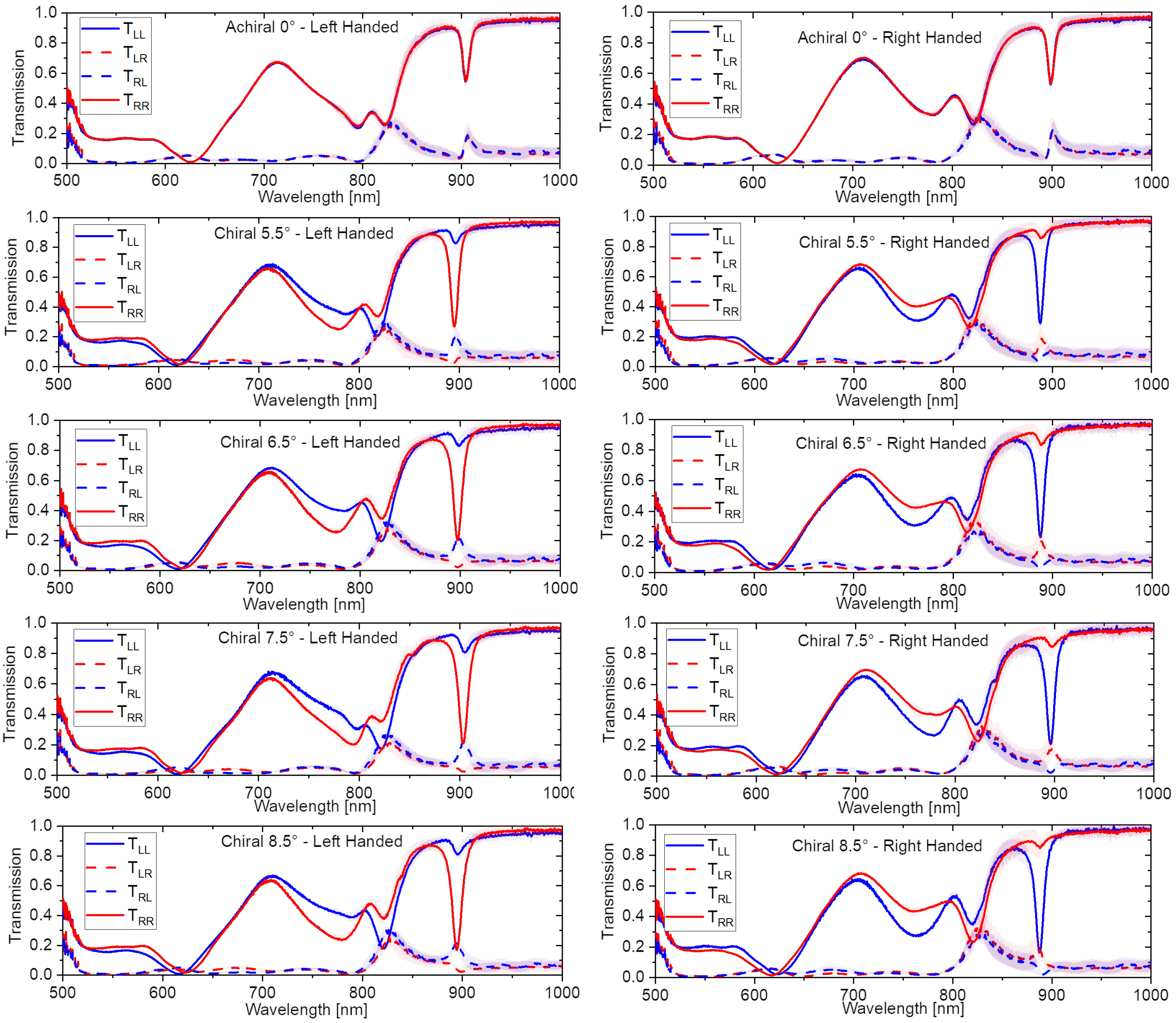

Figure S12: All experimental spectra of the achiral and chiral qBIC metasurfaces with opening angles from 0-8.5°.

Left: Left-handed structures. Right: Right-handed structures. The opening angle increases from top to bottom. All metasurfaces were measured with a chiral analyzer. The solid blue and red lines represent the co-polarization terms, T_{LL} and T_{RR}, respectively, whereas the dashed blue and red lines represent the cross-polarization terms, T_{LR} and T_{RL}, respectively. The solid and dashed lines are the median of 4 measurements (see Figure S15 for more details). The shaded area represents the standard deviation of these 4 measurements.

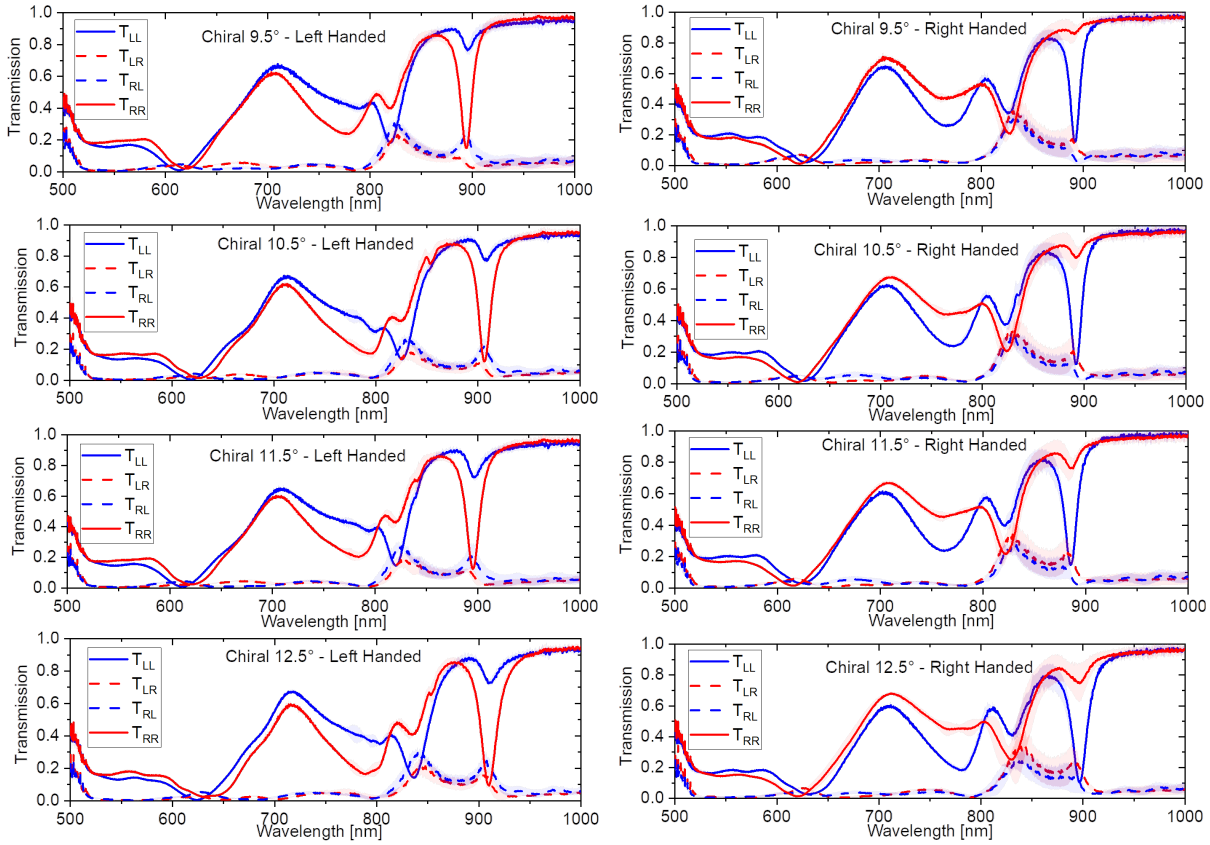

Figure S13: All experimental spectra of the achiral and chiral qBIC metasurfaces with opening angles from 9.5-12.5°.

Left: Left-handed structures. Right: Right-handed structures. The opening angle increases from top to bottom. All metasurfaces were measured with a chiral analyzer. The solid blue and red lines represent the co-polarization terms, T_{LL} and T_{RR}, respectively, whereas the dashed blue and red lines represent the cross-polarization terms, T_{LR} and T_{RL}, respectively. The solid and dashed lines are the median of 4 measurements (see Figure S15 for more details). The shaded area represents the standard deviation of these 4 measurements.

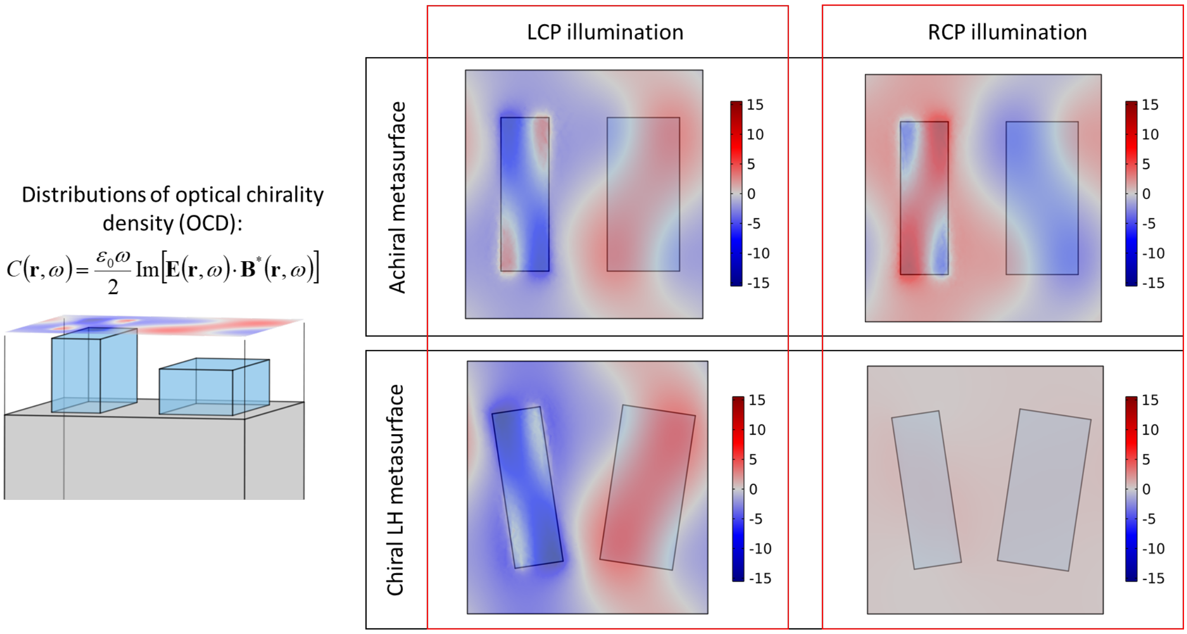

Figure S14: Distributions of optical chiral density (OCD) for the achiral and the chiral left-handed metasurface.

Left: Schematic of the unit cell. The OCD was evaluated 15 nm above the highest rod and normalized by the OCD of the incident wave. Right side: Distribution of OCD for the achiral (top) and chiral LH metasurface (bottom) for LCP (left) and RCP (right) illumination. While the achiral metasurface shows OCD with similar magnitude for LCP and RCP illumination, the chiral LH metasurface shows strong OCD for LCP and contrary no OCD for RCP illumination.

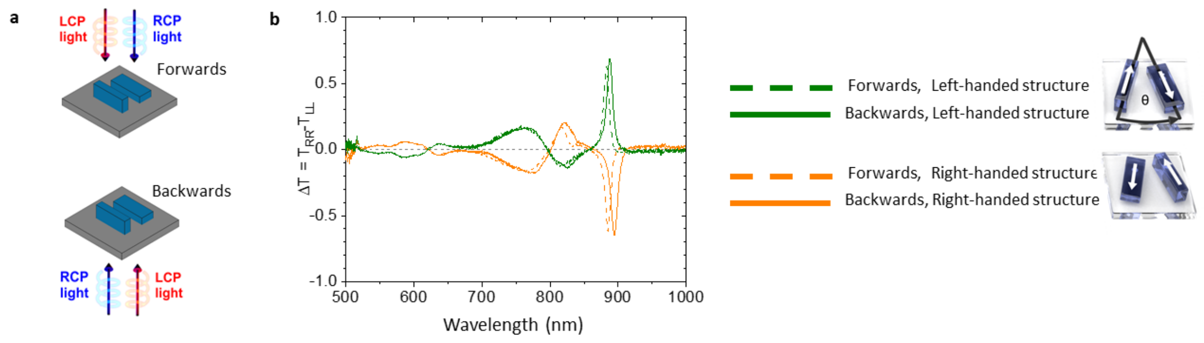

Figure S15: Lorentz reciprocity of the chiral qBIC.

a, Schematic illustration showing the flipping of the substrate to measure it in forwards (solid line) and backwards (dashed line) direction. **b,** Transmittance difference for the right-handed (orange) and left-handed (green) qBIC metasurfaces. The transmittance difference signal of the qBIC metasurfaces does not flip due to their three-dimensionality, contrary to planar chiral structures.

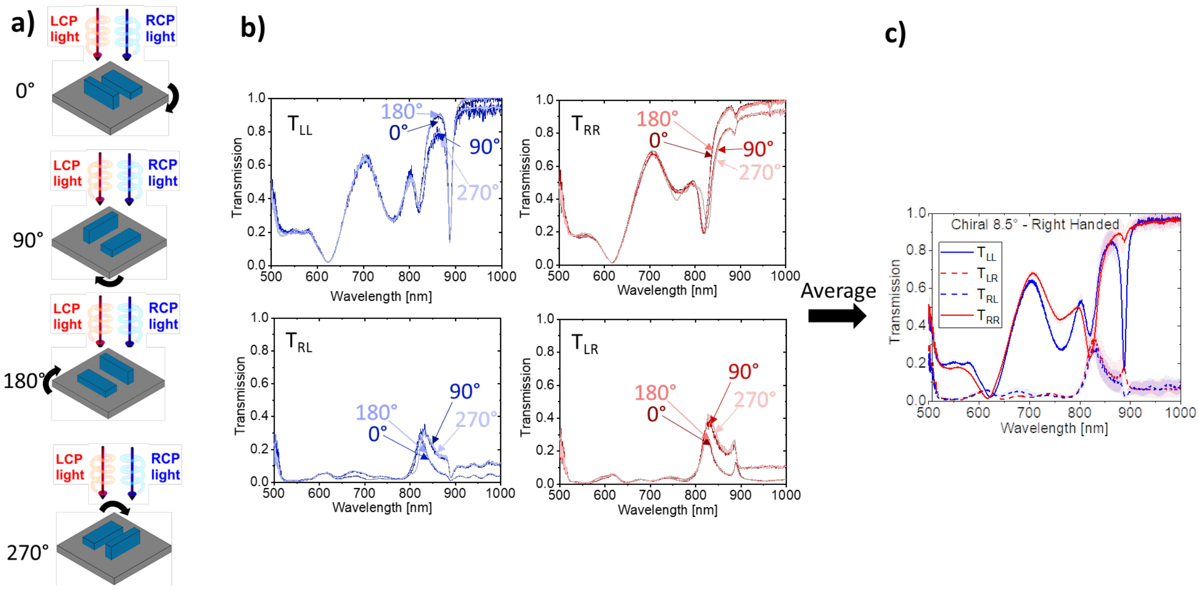

Figure S16: Influence of sample rotation on the measurements of the chiral qBIC metasurfaces with chiral analyzer.

a, Schematic overview of the performed sample rotations during measurement. The sample was rotated four times with a 90° angle to avoid any influences of elliptical polarization or sample tilt. **b, c,** Single spectra of the four measurements (**b**) and the mean value with standard deviation (**c**) of the right-handed chiral qBIC metasurfaces with an opening angle of $\theta = 8.5°$ measured with chiral analyzer. A small signal variation in the transmittance data upon sample rotation is evident in the four single spectra for each co- and cross-polarization (T_{LL}, T_{RR}, T_{LR}, T_{RL}). The signal is identical for rotations of 180° (0°, 180° and 90°, 270°), which is a hint towards a small degree of elliptical polarization, which decreases the measured CD. For an influence of sample tilt, the signal should vary for all rotation angles. The small degree of elliptical polarization might be caused by the deviation of retardation (+/-6%) given by the quarter wave plate (QWP, RAC4.4.20 from B-Halle, 500-900 nm) and by deviation of the chiral analyzer (QWP, AQWP05-580 from Thorlabs, 350-850 nm; polarizer WP25M-UB from Thorlabs, 250-4000 nm).

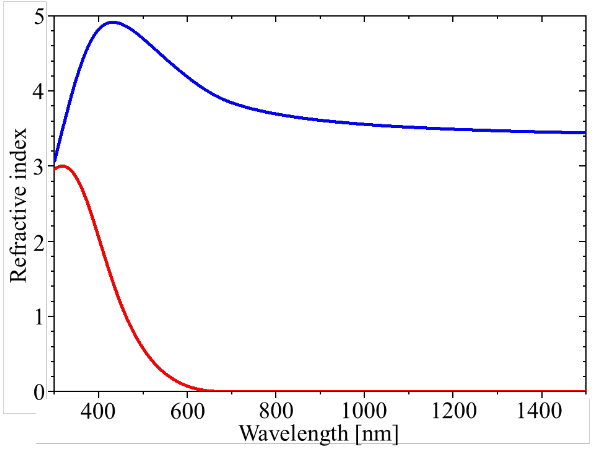

Figure S17. Refractive index of the amorphous silicon.

Real (blue) and imaginary (red) part of the refractive index as determined by spectral ellipsometry. The amorphous silicon was deposited using plasma-enhanced chemical vapor deposition (PE-CVD) from silane (SiH_4) at 250°C.
